# Modelling the Habitat Distribution of the Endemic Azooxanthellate Coral *Madracis interjecta* From the Mesophotic to the Deep Red Sea

**DOI:** 10.1002/ece3.71456

**Published:** 2025-05-22

**Authors:** Megan K. B. Nolan, Pauline Falkenberg, Fabio Marchese, Marta A. Ezeta Watts, Natalie Dunn, Laura Macrina, Viktor Nunes‐Peinemann, Giovanni Chimienti, Silvia Vimercati, Tullia I. Terraneo, Mohammed Qurban, Vincent Pieribone, Carlos M. Duarte, Francesca Benzoni

**Affiliations:** ^1^ Division of Biological and Environmental Science and Engineering King Abdullah University of Science and Technology Thuwal Saudi Arabia; ^2^ Marine Science Program King Abdullah University of Science and Technology Thuwal Saudi Arabia; ^3^ Department of Biosciences, Biotechnology and Environment University of Bari Aldo Moro Bari Italy; ^4^ National Center for Wildlife Riyadh Saudi Arabia; ^5^ OceanX New York USA

**Keywords:** bioherm, deep sea, habitat suitability models, *Madracis interjecta*, mesophotic zone, red Sea

## Abstract

The coral genus *Madracis* has a global distribution from shallow waters to over 1200 m depth. In the Red Sea, the azooxanthellate endemic species *Madracis interjecta* is known to occur from depths of 120 to 350 m. This species is often observed in mesophotic ecosystems and has been reported to form sediment‐binding bioherms, yet the conditions required for these formations are not understood. Here, we extracted quantitative data from video footage to identify the distribution of *M. interjecta* for the first time along the Saudi Arabian Red Sea coast. We present a habitat suitability model to identify potential habitats in the northern Red Sea and Gulf of Aqaba for this species. Combining presence data with geomorphometric variables and environmental data, we identified both depth and seafloor ruggedness as main drivers of this species distribution. Through multivariate statistics, we found that bioherms were found in deeper and cooler waters than individual *M. interjecta* colonies. Due to the narrow continental shelf and steep slopes of the northern Red Sea and Gulf of Aqaba, the effects of coastal development are threatening shallow, mesophotic and deep ecosystems. This work provides both a baseline survey and predicted distributions of an important habitat‐forming scleractinian coral, which can inform conservation planning in the region.

## Graphical abstract

The azooxanthellate Scleractinian *Madracis interjecta* is often observed in mesophotic ecosystems and has been reported to form sediment‐binding bioherms, yet the conditions required for these formations are not understood. We present a habitat suitability model to identify potential habitats in the northern Red Sea and Gulf of Aqaba for this species and through multivariate statistics find that bioherms are present in deeper and cooler waters than individual *Madracis interjecta* colonies.

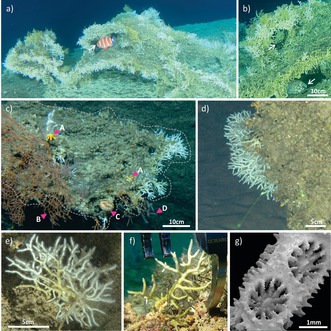

## 1 Introduction

Broadly, species distributions are determined by local environmental and geomorphological conditions, species physiology, dispersal mechanisms and biotic interactions (Bozinovic et al. 2011; Evans et al. 2015; Ramos et al. 2016; Wisz et al. 2013). Climate change and local anthropogenic impacts are influencing the distributions of marine species (Hastings et al. 2020; Martello et al. 2024; Wernberg et al. 2024), altering environmental conditions and therefore affecting community compositions (Harley et al. 2006; Pinsky et al. 2013). To predict the responses of species to climate change, a comprehensive understanding of the species' current distribution, their population's connectivity, and their ecological niches is required (Crowder and Norse 2008). The inaccessibility of the mesophotic and deep sea, which lies below conventional SCUBA diving limits, means that they receive less attention (Jamieson et al. 2020; Kahng et al. 2014; Loya et al. 2016; Pyle 1996) and the biology and ecology of their key organisms remain largely unknown (Bongaerts et al. 2010; Kahng et al. 2010; Waller et al. 2023). Furthermore, the remoteness of deeper marine environments makes it challenging to conduct surveys on the abundance and distribution of species across meaningful scales (Armstrong et al. 2019).

Expanding the focus of such research to mesophotic (approximately 30–150 m) and deep (> 150 m) zones requires a major leap in our understanding of the processes and environmental conditions shaping the form and function of mesophotic and deep‐sea coral ecosystems (De Clippele et al. 2020; Eyal et al. 2016; Loya et al. 2016). Bioherms are mound‐like formations of biological origin, such as coral, creating mesohabitats from the mesophotic to the deep sea (Greene et al. 1999; Roberts et al. 2008), via the build‐up and diagenesis of organisms (Ingrosso et al. 2018). These structures consist of both extant and fossil features formed by biological and geological processes leading to a positive seafloor topographical relief, comparable to biogenic reefs found in shallow and photic tropical marine ecosystems (McMannus 2001) and temperate waters (Enrichetti et al. 2019; Roncolato et al. 2024). Considerable advances have been made over the last decade to understand how and where deep biogenic reefs such as coral mounds or bioherms form. For example, changes in dissolved oxygen concentration over geological timescales were revealed as a potential driver of mound formation in a cold‐water coral mound in the Atlantic Ocean (Wienberg et al. 2018). Furthermore, recent research has improved our understanding of the role of hydrodynamics in coral mound formation; both large‐scale currents and small‐scale internal tides may initiate mound formation, while annual up‐ and downwellings have been shown to influence benthic zonation (van der Kaaden et al. 2024, 2021; Wienberg et al. 2020).

In the Red Sea, abiotic conditions below the euphotic zone are unique compared to other basins, due to higher temperatures (remaining above 21°C even at 3000 m) and salinities (> 40 PSU below the mesophotic; Berumen, Voolstra, et al. 2019; Roder et al. 2013). Under these conditions, and specifically between 120 and 170 m depth, coral‐dominated ecosystems are well‐developed and often present high benthic diversity (e.g., Fricke and Knauer 1986; Kramer et al. 2019; Terraneo et al. 2023; Vimercati et al. 2024). One of the known components of both mesophotic and aphotic reefs in the Red Sea is scleractinian corals of the genus *Madracis* Milne Edwards & Haime, 1849. On a global scale, *Madracis* is found from tropical to temperate waters (Benzoni et al. 2018; Diekmann 2003; Morri et al. 2000; Santodomingo et al. 2007; Scheer and Pillai 1983), and is eurybathic, with the deepest known record at 1220 m in the tropical Atlantic for *Madracis myriaster* (Milne Edwards & Haime, 1850) (Cairns 2000; Reyes et al. 2010; Roberts 2009). In the Red Sea, two *Madracis* species have been reported to date: the facultative symbiotic *Madracis kirbyi* Veron & Pichon, 1976, distributed throughout the photic and mesophotic Indo‐Pacific (Benzoni et al. 2018); and the azooxanthellate *Madracis interjecta* Marenzeller (1907), found endemically in the mesophotic and aphotic zones of the Red Sea. The former grows in relatively small (< 10 cm in largest diameter) encrusting to submassive colonies and, following the classification by Roberts (2009, 24), can be considered functionally non‐constructional and ahermatypic in the Red Sea. Conversely, *M. interjecta* is known to form finely branching colonies that can reach up to 30 cm in largest diameter, and are constructional and hermatypic (*sensu* Schuhmacher and Zibrowius 1985). Furthermore, *M. interjecta* is known to form sediment‐binding bioherms from 120 m to 350 m (Fricke and Hottinger 1983). Thus far only reported from the northern Gulf of Aqaba, these bioherms modify the topographical relief on the underlying seafloor (Fricke and Hottinger 1983). Moreover, they provide a microhabitat for their diverse associated fauna, including brachiopods, sponges and cirripeds (Fricke and Hottinger 1983). *Madracis* bioherms therefore represent an enigmatic and understudied biological and sedimentological feature of the mesophotic and aphotic zones of the Red Sea. Their occurrence has never been reported outside the Gulf of Aqaba, and the environmental conditions conducive to their establishment and growth are largely unknown.

In order to better understand the distribution of bioherm‐forming species, such as *M. interjecta* , habitat suitability models (HSMs) can be used (Elith and Leathwick 2009). In other ecosystems, these models have informed active management, including the establishment of marine protected areas (MPAs; Vaughan and Agardy 2019) and restoration projects, which have predominantly focused on shallow water ecosystems, found above 30 m depth (e.g., Asaad et al. 2018; da Silveira et al. 2021; Nolan et al. 2021; Suggett and van Oppen 2022). Although several important deep‐sea ecosystem services have been identified (La Bianca et al. 2023; Thurber et al. 2014), the active management of deeper ecosystems is lagging behind that of shallow, photic ecosystems (Soares et al. 2020), highlighting the need for species distribution models in the mesophotic and deep. In the Saudi Arabian Red Sea in particular, there are no MPAs which extend into deep waters (ncw.gov.sa), despite the abundance of biodiversity at greater depths (e.g., Fricke and Knauer 1986; Kramer et al. 2019; Terraneo et al. 2023; Vimercati et al. 2024). Much of the species distribution data collected from the deep sea is presence only data, as true absences are unreliable (Vierod et al. 2014). There are several modelling techniques that use background or pseudo‐absences in the place of true absences, such as boosted regression trees (Elith et al. 2008). One of the best performing algorithms is maximum entropy (MaxEnt) (Elith et al. 2006; Tittensor et al. 2009), which has previously been successfully applied to deep‐sea coral species (e.g., Howell et al. 2011; Tittensor et al. 2009).

Here, we aim to understand the distribution of *M. interjecta* colonies and bioherms along the whole Saudi Arabian Red Sea coast, spanning 12.7° of latitude. We extract and describe this distribution, and then use a MaxEnt HSM to predict the species habitat distribution in the northern Red Sea and Gulf of Aqaba, an area for which high‐resolution, continuous bathymetric data is available. This allowed us to characterise the physical and environmental space in which *M. interjecta* survives both as an individual colony and where it is able to form bioherms.

## 2 Methods

### 2.1 Acoustic Data

This study was conducted in the Saudi Arabian waters of the Red Sea, a young ocean basin with limited connectivity through the Bab‐el‐Mandeb to the Gulf of Aden and wider Indian Ocean (Augustin et al. 2021; Wang et al. 2019). This relative isolation, combined with very little freshwater input, has resulted in a very warm and saline basin (Roder et al. 2013). Extensive exploration of this region was conducted during three expeditions on board M/V OceanXplorer1: the Deep Blue expedition in 2020, the Red Sea Decade Expedition (RSDE) in 2022, and the Red Sea Relationship Cultivation (RSRC) mission in 2022.

During all expeditions, M/V OceanXplorer1 was equipped with a Kongsberg EM304 multibeam echosounder to collect bathymetric and backscatter data down to 3000 m depth. Sound velocity profiles were obtained with eXpendable BathyThermographs (XBTs) and were used to calibrate the bathymetric acquisition. The bathymetry and backscatter were acquired with QPS Qinsy and processed to 30 m resolution in QPS Qimera and QPS FMGT, respectively. Acoustic data were collected from an area of almost 85,000 km^2^ between the Saudi Arabian Red Sea coastline and the central axis, and from the northern Gulf of Aqaba (29.4° N) to the southern Red Sea (16.5° N).

For the Habitat Suitability Models (HSMs), data from the Deep Blue expedition in 2020 was used, covering a total area of 33,970 km^2^ in the northern Red Sea and Gulf of Aqaba. The focus area for HSMs was chosen due to the acquisition of contiguous bathymetric and backscatter data, the density of video surveys and the regional importance for the giga‐project NEOM (neom.com; Berumen, Roberts, et al. 2019). From this bathymetric dataset, 12 geomorphometric explanatory variables were extracted using default algorithms in ArcMap v10.8 and Saga v8.1.1. Aside from water depth, the extracted parameters were aspect (both Northness and Eastness; Horn 1981), slope (Zevenbergen and Thorne 2006), Bathymetric Position Index (BPI; Weiss 2001), Convergence Index (CI; Koethe and Lehmeier 1996), local Convexity Index (CX; Iwahashi and Pike 2007), surface area to planar area ratio (rugosity; Jenness 2004), Terrain Surface Texture (TEX; Iwahashi and Pike 2007), Vector Ruggedness Measure (VRM; Sappington et al. 2007), mean curvature, planar curvature and profile curvature (Zevenbergen and Thorne 2006). For several parameters (BPI, CI, CX, TEX, VRM), different window sizes were initially included to determine the most appropriate scale at which to include these parameters. These parameters were selected to represent components of seafloor geomorphology that influence species distributions as in Bargain et al. (2017). Where available (i.e., where bathymetric data overlapped with transects), geomorphometric parameters were also extracted from bathymetry for the rest of the study area in the Red Sea for comparison between the environmental space of *M. interjecta* colonies and bioherms (See below: *Statistical Analysis*).

### 2.2 Environmental Data

To measure environmental conditions, an RBR*maestro* ^*3*^ logger was attached to either the Remotely Operated Vehicle (ROV) or submersibles during dives, with sensors to continuously measure temperature (Marine CT), salinity (Marine CT 2000 m) and dissolved oxygen concentration (RBRcoda T.ODO|fast) throughout the whole study area. In the northern Red Sea and Gulf of Aqaba, this provided us with continuous data at near‐bottom depths. Using the Data‐Interpolation Variational Analysis (DIVA) function of Ocean Data View v5.6.1 (Schlitzer 2021) and Inverse Distance Weighted (IDW) interpolation in ArcMap, we modeled salinity, temperature and oxygen concentration across the focus area for HSMs (see methods in Nolan et al. (2024) for further details on layer creation and justifications).

### 2.3 Sampling, Identification and Video Analysis

Georeferenced video imagery was collected during the three aforementioned expeditions using cameras mounted onto an ROV (Chimaera, CHR) and two manned submersibles (Neptune, NTN and Nadir, NDR) over a total of 278 dives, generating over 1335 h of high‐resolution footage. The ROV was an Argus Mariner XL work class ROV, and the submersibles were both Triton 3300/3. CHR was equipped with several cameras, including HDTV 1080p and Arctic EagleRay 4 K cameras, as well as a Kongsberg HIPaP 501 USBL (Ultra‐Short BaseLine). NTN had an Arctic EagleRay 4 K camera, and NDR had a Wide‐Angle Helium 8 K Canon and macro Helium 8 K Nikon cameras. Both submersibles were equipped with a Sonardyne Ranger Pro 2 USBL. Hydraulic manipulator arms were also used onboard CHR and NTN for sample collection.

**FIGURE 1:**
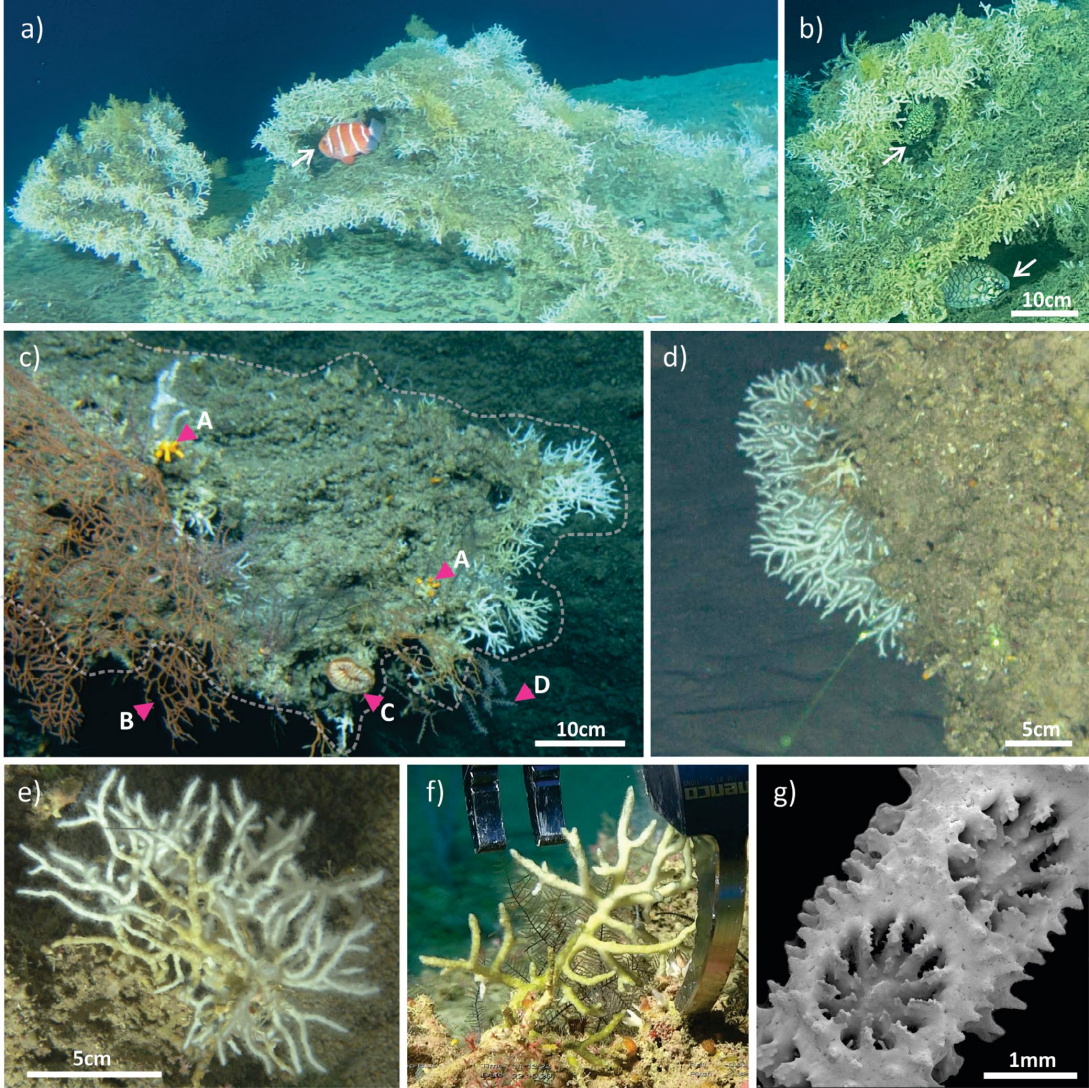
*Madracis interjecta* bioherms and colonies. *Madracis interjecta* bioherms (a–c) and colonies (d–f) in situ, and scanning electron microscope (SEM) imagery of the corallites for identification. (a) A monospecific *M. interjecta* bioherm at 275 m (NTN0055), showing the large scale structure, and the shelter provided for *Pristigenys refulgens* indicated by the arrow, (b) the same *M. interjecta* bioherm 275 m (NTN0055), providing shelter to two *Monocentris japonica* individuals, indicated by arrows, (c) a large colony, the structure indicated by a dashed line, mostly dead at the base and already inhabited by associated benthos indicated by the arrowheads, including A‐ *Dendrophylliidae* sp., B‐ *Melithaea* sp., C‐ *Rhizotrochus typus* , and D‐ *Acanthogorgiidae*, 163 m depth (CHR0043), (d) a colony growing on a ledge, 171 m (NTN0037), (e) a colony at 166 m (NTN0035), (f) a colony at 147 m (CHR0019) being collected for identification and genetic analyses, and (g) SEM imagery showing the corallites along a branch of sample NTN0035_9_01, collected from 167 m.

Specimens of *M. interjecta* were collected during ROV and submersible dives, assigned a specimen voucher, and dried. Species‐level identification was performed through comparison of the collected specimens skeletal morphology and the original descriptions and illustrations (Plate 2, Figure 3 within reference, Figure 1; Marenzeller 1907). Their skeletal features were analysed with Scanning Electron Microscopy (SEM) imagery performed at the KAUST Imaging and Characterisation Core Lab following the protocol in Terraneo et al. (2019).

The video footage was analysed to identify and record the presence of *M. interjecta* colonies based on the in vivo morphology of the collected, identified specimens. We recorded the presence of *M. interjecta* for each new, non‐overlapping frame of the video, regardless of the number of individual colonies seen in the field of view (hereafter termed ‘observations’). Additionally, the growth form of the observation was recorded, determining whether *M. interjecta* was growing as a single colony or as a bioherm. When bioherms were composed of both live and dead skeleton (e.g., Figure 1c), and the presence of the live *M. interjecta* colony was in structural continuity with the dead portion that formed the bioherm's main framework, we were able to confidently identify these as *Madracis* bioherms. Following examination of all transects, those which did not record any benthic video in the identified depth range of the species (here, above 300 m) were excluded from further descriptive statistics.

### 2.4 Model Development and Validation

**FIGURE 2:**
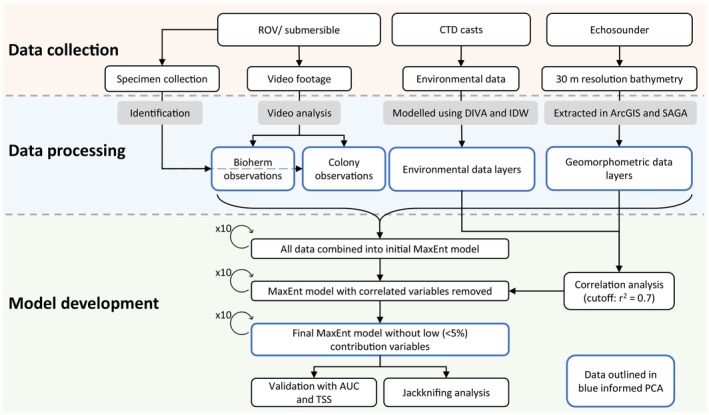
Flowchart highlighting the main steps of the methodology, separated into data collection (orange background), data processing (blue background) and model development (green background). Blue outlines indicate data that was then used for the Principal Component Analysis (PCA). AUC, area under the receiver operating characteristics curve; CTD, conductivity, temperature, depth sensor; DIVA, data‐interpolation variational analysis; IDW, inverse distance weighted; ROV, remotely operated vehicle; TSS, true skills statistic. r2 is the calculated Pearson's correlation coefficient.

Models were developed to predict the suitability of habitat in which *M. interjecta* may occur. To run the HSMs, the software MaxEnt v3.4.4 was used with presence only data from the *M. interjecta* observations, and geomorphological and oceanographic predictor variables in raster form at a resolution of 30 m. Following spatial thinning in MaxEnt to reduce errors arising from spatial autocorrelation, one presence record was kept in each 30 m^2^ cell, resulting in a sample of 81 observations was used to run the model, with 70% of the data (*n* = 57) used for training, and the remaining 30% (*n* = 24) for testing. The models were run initially with all variables included, using all default settings aside from the following: only hinge features were enabled, 10 runs were carried out using bootstrapping, and a regularisation multiplier of 2.5 was used for optimal smoothing of the model. These parameters were selected based on trials with preliminary data, based on the best performing models according to the Area Under the receiver operating characteristics Curve (AUC) value, as well as the work conducted by Nolan et al. (2024). Cross‐validation was also tested as an alternative validation method, but provided similar results to bootstrapping, so only one method is presented here. A correlation analysis was run between all variables and a cut off value of *r* ^2^ = 0.7 was implemented, using the percent contribution to the model to determine the best variable to keep among those that were strongly correlated. The MaxEnt model was then rerun with this reduced set of predictor variables (Table 1). The percent contribution of these variables was analysed, and any variables with a contribution less than 5% was removed, and the model was re‐run. This process ensures that the final model is as parsimonious as possible. Jackknifing was enabled, meaning that the model was also run with each variable removed in turn, as well as with each variable alone, allowing a better understanding of the contribution of each variable, and the potential redundancy between variables. The AUC was used to assess the performance of the models (Merow et al. 2013). Additional validation of the model was provided with the True Skills Statistic (TSS), which was calculated using the MaxSSS (Maximising the Sum of Sensitivity and Specificity) as the threshold (Allouche et al. 2006; Liu et al. 2016). The methodological workflow is outlined in Figure 2.

**TABLE 1:** Ten variables used in the final habitat suitability models, the cell neighbourhood size for analysis, and their percent contribution to the MaxEnt model results.

Variable (units)	Neighbourhood size	Percent contribution	Algorithm reference
Depth (m)	—	43.1	—
Vector Ruggedness Measure	5	25.6	(Sappington et al. 2007)
Rugosity (surface area: planar area ratio)	3	16.2	(Jenness 2004)
Terrain Surface Texture	5	8.4	(Iwahashi and Pike 2007)
Dissolved Oxygen Concentration	—	6.8	—

### 2.5 Statistical Analysis

The environmental and geomorphometric conditions in which individual colonies and bioherms were observed were assessed visually and through a Principal Component Analysis (PCA) in R v4.4.0 (Core Development Team 2020), using the packages vegan v2.6.6 (Oksanen et al. 2024), FactoMineR v2.11 (Lê et al. 2008) and factoextra v1.0.7 (Kassambara and Mundt 2020). The analysis was based on uncorrelated variables used in the second habitat suitability model (Figure 2). Due to the patchiness of acoustic data acquisition, some observations do not have overlapping geomorphometric data. These observations without a complete set of variables were removed, resulting in a dataset of 199 observations for the PCA: 142 in the Gulf of Aqaba, 47 in the northern Red Sea, one in the central Red Sea, and nine in the southern Red Sea.

## 3 Results

### 3.1 Specimen Collection and Identification

**FIGURE 3:**
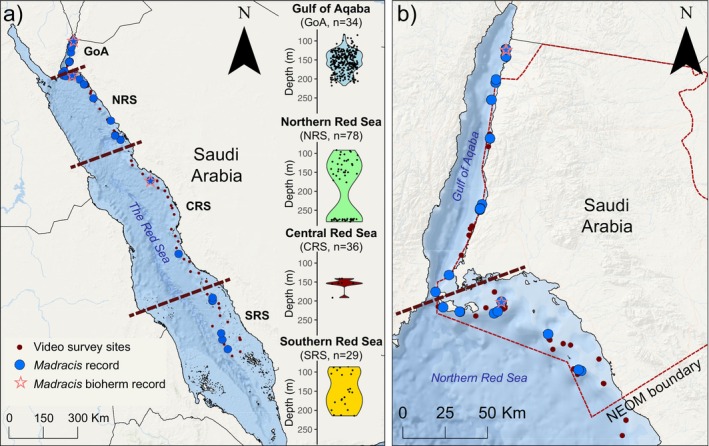
Sampling effort and observed occurrence of *Madracis interjecta* . Sampling along the 362 ROV and submersible video transects above 300 m along (a) the Saudi Arabian Red Sea coast and (b) the northern Red Sea and Gulf of Aqaba (dashed line indicates the NEOM territory jurisdiction). Panels on the right‐hand side in (a) are the violin plots showing the species observation density along the depth gradient at each of the four Red Sea regions, namely the Gulf of Aqaba (GoA), the northern Red Sea (NRS), the central Red Sea (CRS), and the southern Red Sea (SRS), with regions separated by dashed lines in the maps. Colours correspond to those in Figure 6 (GoAblue, NRS–green, CRS–red, and SRS–yellow). The sampling effort (*n*) is shown as the number of transects in the relevant depth range (above 300 m) for each region in panel (a). *M. interjecta* bioherm records are indicated by a star. Basemap sources: ESRI, Garmin, GEBCO, NOAA NGDC, and other contributors.

Covering a latitudinal gradient from 17.2° N to 29.2° N, we collected 30 specimens of *Madracis interjecta* (Figure 1): 10 from the Gulf of Aqaba, 14 from the northern Red Sea, two from the central Red Sea, and four from the southern Red Sea. Specimens were collected between 96 m and 278 m depth (Table 2). From a total of 177 video transects, we identified the presence of *M. interjecta* (Figure 1) in the Saudi Arabian waters of the Red Sea 392 times in 42 transects, with a depth range from 83 m to 280 m (mean = 160.7 m; Figure 3). Colonies were observed along 15 transects (44%) in the Gulf of Aqaba, 17 (22%) in the northern Red Sea, two transects (6%) in the central Red Sea, and eight transects (28%) in the southern Red Sea (Figure 3). Colonies were identified from a range of habitats, but most commonly associated with a steep to vertical seafloor.

**TABLE 2:** Collected specimens of *Madracis interjecta* , detailing the sample ID, dive code, depth, and region for each specimen. In two cases, the sample was bycatch from the dive, and depth is unknown.

Sample ID	Dive code	Depth (m)	Region
CHR0019‐7	CHR0019	147	Gulf of Aqaba
CHR0043‐38B	CHR0043	*Unknown*	Gulf of Aqaba
NTN0035‐9	NTN0035	167	Gulf of Aqaba
NTN0060‐8A	NTN0060	154	Gulf of Aqaba
NTN0170_2B	NTN0170	157	Gulf of Aqaba
NTN0170_2C	NTN0170	157	Gulf of Aqaba
NTN0170_3B	NTN0170	153	Gulf of Aqaba
NTN0170_9B	NTN0170	130	Gulf of Aqaba
NDR0916_8	NDR0916	129	Gulf of Aqaba
NTN0178_7B	NTN0178	277	Gulf of Aqaba
CHR0038‐16	CHR0038	119	Northern Red Sea
NTN0055‐3	NTN0055	278	Northern Red Sea
NTN0055‐4B	NTN0055	278	Northern Red Sea
NTN0055‐5	NTN0055	277	Northern Red Sea
NTN0144‐BIO6	NTN0144	145	Northern Red Sea
NDR0919_6A	NDR0919	96	Northern Red Sea
NDR0920_1B	NDR0920	150	Northern Red Sea
NDR0920_2B	NDR0920	150	Northern Red Sea
NDR0920_3D	NDR0920	159	Northern Red Sea
NDR0920_8B	NDR0920	131	Northern Red Sea
NDR0920_9B	NDR0920	122	Northern Red Sea
NDR0920_12D	NDR0920	113	Northern Red Sea
NTN0174_2G	NTN0174	130	Northern Red Sea
NTN0174_4B	NTN0174	128	Northern Red Sea
CHR200‐BIO22	CHR0200	191	Central Red Sea
NTN0151‐BIO23	NTN0151	141	Central Red Sea
CHR0232BIO26	CHR0232	200	Southern Red Sea
CHR0235BIO7B	CHR0235	214	Southern Red Sea
NTN0134_bycatch	NTN0134	Unknown	Southern Red Sea
CHR0241BIO7C	CHR0241	152	Southern Red Sea

### 3.2 Bioherm Observations

Three *M. interjecta* bioherms were observed. The largest bioherm was recorded in the northern Gulf of Aqaba (transect NTN0160), forming a continuous three‐dimensional structure of live and dead *M. interjecta* , which continued for approximately 150 m in length, and upslope from 215 m to 143 m depth. Another large *Madracis* bioherm was observed in the northern Red Sea (transects NTN0055, NDR0925 and NTN0178; Figure 1a,b), between 271 m and 280 m depth (diameter > 60 m), and a smaller bioherm at 150 m depth (diameter < 5 m) in the central Red Sea (transect NTN0151). We also recorded and identified associated organisms, although our observations from the ROV and submersible videos were limited to organisms larger than approximately 1 cm in height and did not allow us to determine the smaller components of the benthic assemblages growing on the *M. interjecta* bioherms. Among the larger taxa, octocoral genera such as *Melithaea* Milne Edwards, 1857 and *Acanthogorgia* Grey, 1857 (Figure 1c) and scleractinians of the family Dendrophylliidae Grey, 1847 (Figure 1c) and the solitary *Rhizotrochus typus* Milne Edwards & Haime, 1848 (Figure 1c) were commonly associated with *M. interjecta* bioherms. Fish were also observed utilizing these structures for shelter, including *Pristigenys refulgens* (Valenciennes, 1862) (Figure 1a) and *Monocentris japonica* (Houttuyn, 1782) (Figure 1b), both of which show a widespread Indo‐Pacific distribution (Iwatsuki et al. 2012; Su et al. 2022).

### 3.3 Habitat Suitability Modelling

The HSM was generated to predict the areas in which environmental conditions are potentially fitting for *M. interjecta* over 33,970 km^2^ of seafloor in the northern Red Sea and Gulf of Aqaba. The model was validated with the AUC values (Merow et al. 2013), which were extremely close to one, at 0.9956 and 0.9950 for training and test data respectively, providing us with high confidence in these results. These AUC values are also close to each other, an indication that there was no detrimental overfitting of the model to the training data. The results of the TSS analysis provide a threshold of model confidence from −1 (complete disagreement) to +1 (complete agreement) (Allouche et al. 2006; Liu et al. 2016). Here, the average TSS score across 10 bootstrapping runs was 0.9702 (range: 0.9054–0.9902), indicating a well performing model. Following the removal of any correlated variables (r^2^ > 0.7), the final model was generated with five variables: depth, VRM, seafloor rugosity, TEX and dissolved oxygen concentration (Table 1). Depth was the most informative predictor, contributing just over 41% to the model, followed by VRM, which contributed 25.6% (Table 1). Jackknife analysis revealed that depth contains the most unique information, as it resulted in the best single‐variable model. Despite this, the test AUC did not fall by more than 0.0007 when any single variable was removed, suggesting some potential redundancy among predictors.

**FIGURE 4:**
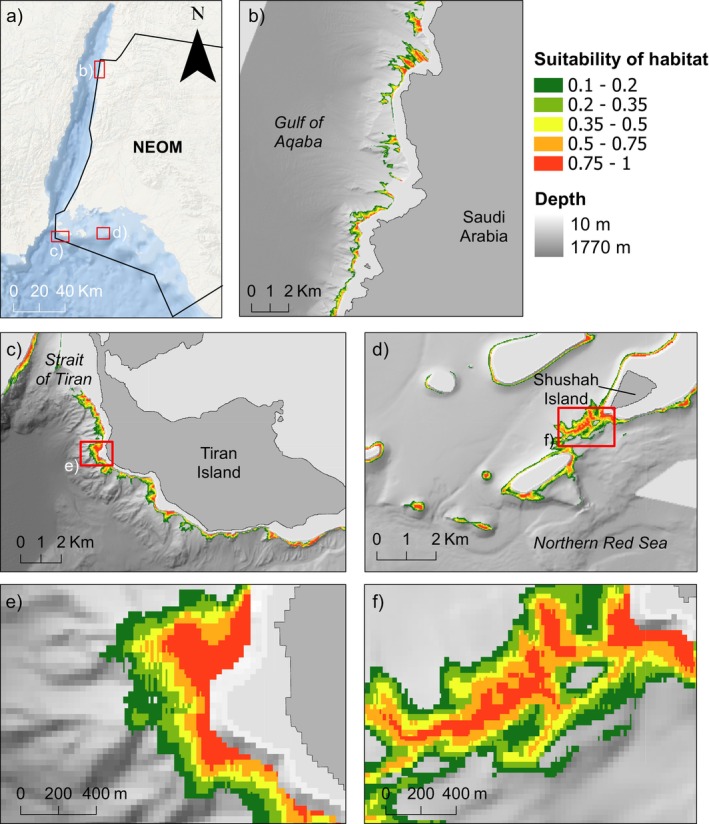
Maps identifying the areas that are highly suitable for *Madracis interjecta* according to the habitat suitability model. Map of the overall target area for habitat suitability modelling, with the jurisdiction of NEOM shown by a white outline, and focus areas (b–d) by red boxes (a). The results of habitat suitability models for the northern Gulf of Aqaba (b), south of Tiran Island (northern Red Sea) with a red box indicating focus area (c), and the area around Shushah Island in NEOM (northern Red Sea) with a red box indicating focus area (d). High resolution areas are shown in (e) and (f). The highest suitability (with a probability of the habitat being suitable above 0.75) is shown in red, and data between 0 and 0.1 is masked for visualisation. Basemap sources for panel (a): ESRI, Garmin, GEBCO, NOAA NGDC and other contributors.

**FIGURE 5:**
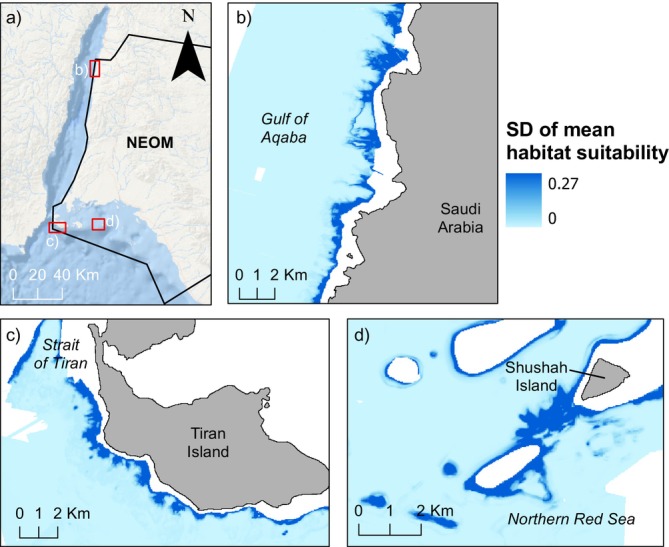
Standard deviation (SD) of habitat suitability model results. Map of the overall target area for habitat suitability modelling, with the jurisdiction of NEOM shown by a black outline, and focus areas (b–d) by red boxes (a). The standard deviation (SD) of the mean habitat suitability from ten bootstrapping runs for the northern Gulf of Aqaba (b), south of Tiran Island (northern Red Sea) (c), and the area around Shushah Island in NEOM (northern Red Sea) (d). The highest SD (0.27) is shown in darker blue. Basemap sources for panel (a) ESRI, Garmin, GEBCO, NOAA NGDC, and other contributors.

Areas indicated by MaxEnt to have a probability of being suitable > 0.75 were considered to be highly suitable, while those with a probability of 0.5–0.75 were considered moderately suitable. The results of the MaxEnt model indicated an area totaling 65.84 km^2^ in the Red Sea Exclusive Economic Zone of Saudi Arabia that was highly suitable (> 0.75) for *M. interjecta* (Figure 4), representing 27.05% of the appropriate depth range within the study area. Highly suitable areas were distributed throughout the northern Red Sea and Gulf of Aqaba, including large patches close to the Saudi Arabian–Jordanian border, and seen intermittently for 31 km south along the coastline (Figure 4b). Large, highly suitable areas were also seen just outside the Strait of Tiran, to the north and south, particularly around Tiran Island (Figure 4c). Another area, of 5.2 km^2^, that was found to be highly or moderately suitable (probability > 0.5) was located close to Shushah Island (Figure 4d). The Standard Deviation (SD) of the mean habitat suitability between the 10 bootstrapping runs was low on average, at 0.002 (Figure 5), with a maximum value of 0.27. Approximately 2.16 km^2^ of the study area had a SD of 0.2–0.27 (0.005% of the whole study area) and a further 104.3 km^2^ had a SD between 0.1 and 0.2 (0.24% of the whole study area).

### 3.4 Statistical Analysis

**FIGURE 6:**
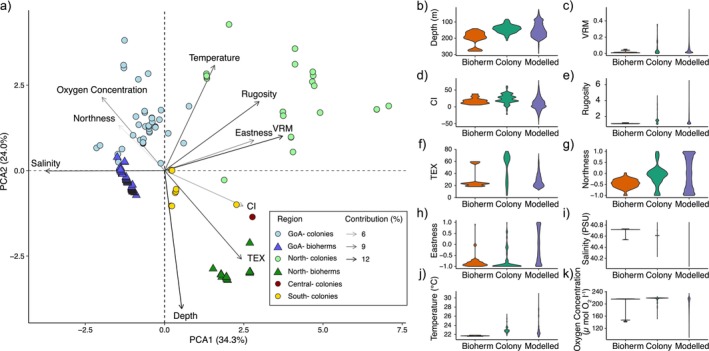
Environmental conditions driving the distribution of *Madracis interjecta* in the Red Sea based on this study. (a) A principal component analysis (PCA) biplot shows the position of *M. interjecta* colony records (circle) and bioherm records (triangle) in the multivariate space represented along PC1 and PC2. The fill colour indicates the four Red Sea regions (Gulf of Aqaba, GoA–light blue for colonies and dark blue for bioherms, northern Red Sea–light green for colonies and dark green for bioherms, central Red Sea–red for colonies, and southern Red Sea–yellow Sfor colonies). No bioherms were included for the central and southern Red Sea. The predictor variables and their percent contribution are shown as labelled arrows: Variables with the lowest contribution (6%) are represented by shorter, light grey arrows, and variables with the highest contribution (12%) are shown by longer, black arrows. (b–k) shows eight variables, with violin plots indicating the density of both observations (separated into bioherm‐forming (red) and individual colonies (green)) and predicted occurrences (probability > 0.75; purple) of *M. interjecta* within the range of each variable. Data for salinity, temperature, and oxygen concentration is restricted to the Northern Red Sea and Gulf of Aqaba. TEX is Terrain Surface Texture, VRM is Vector Ruggedness Measure, and CI is Convergence Index.

**FIGURE 7:**
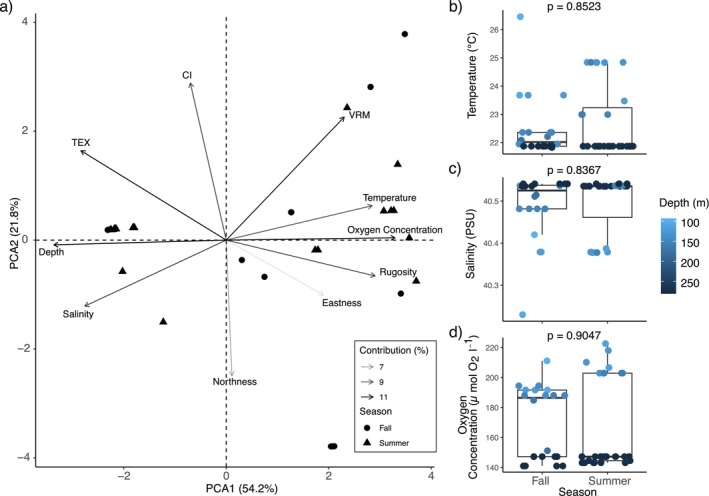
(a) A Principal Component Analysis (PCA) biplot shows the environmental conditions driving the distribution of *Madracis interjecta* records (including all colony and bioherm records combined) in the northern Red Sea. The multivariate space is represented along PC1 and PC2 and driving variables are shown on labelled arrows, with longer and darker arrows representing higher variable contribution (from 7% in grey to 11% in black). Variable data was collected in two seasons, fall and summer, indicated by circles and triangles respectively. The differences between (b) temperature, (c) salinity and (d) oxygen concentration are shown by boxplots and jittered points, coloured by depth. Boxes indicate interquartile ranges with a central line indicating the median value. The whiskers represent 1.5 * the interquartile range, and outliers are not highlighted. The *p* value above each panel indicates the result of a Wilcoxon Rank Test and shows no significant difference between any groups.

The PCA analysis identified the environmental and geomorphometric space that individual colonies and bioherms of *M. interjecta* inhabited (Figure 6). Using all observations for which variables were available (*n* = 199), the first two Principal Components (PC) explained 58.3% of the variation, primarily by salinity and VRM on PC1, and depth and temperature for PC2 (Figure 4a). Data points visually clustered by region, particularly along PC1. The most variation within a region was seen in the northern Red Sea, chiefly along PC2, which is largely influenced by temperature. The northern Red Sea was studied during three expeditions in different seasons across years, including October and November in 2020 (fall), and from April to July of 2022 (summer), so we tested whether season was driving these clusters by performing a PCA with data solely from the northern Red Sea and observed no clustering of *M. interjecta* observations based on the season (Figure 7a). We also observed no significant difference (based on Wilcoxon Rank tests for non‐parametric data) between summer and fall for temperature (Wilcoxon Rank test: w = 279, *p* = 0.8523; Figure 7b), salinity (Wilcoxon Rank test: *w* = 280, *p* = 0.8367; Figure 7c) or oxygen concentration (Wilcoxon Rank test: *w* = 264, *p* = 0.9047; Figure 7d). Within regions, clustering of bioherm observations was also seen (Figure 6a).

In the study area, *M. interjecta* bioherms were found in deeper waters than the single colonies of the same species (Figure 6b). Additionally, the geomorphological area occupied by bioherms was generally more restricted than the area suitable for *M. interjecta* colonies, particularly in measures of seafloor complexity, which were generally lower for bioherms (VRM; Figure 6c and Rugosity, Figure 6e). Furthermore, bioherms were observed in more restricted salinities (40.54–40.73 PSU) than colonies (40.23–40.85 PSU; Figure 6i), as well as more restricted temperatures (21.60°C–21.87°C for bioherms, 21.76°C–26.46°C for colonies; Figure 6j). Dissolved oxygen concentration was more comparable between the two growth morphologies (141.05–217.04 μ mol O_2_ l^−1^ for bioherms, 151.24–224.31 μ mol O_2_ l^−1^ for colonies). For all variables except TEX, models predicted that *M. interjecta* could survive in a broader range of conditions than we observed (Figure 6b–k).

## 4 Discussion

Using video analysis and habitat suitability models, we were able to assess the distribution of the Red Sea endemic coral *M. interjecta* along the Saudi Arabian coastline. Our identifications of colonies in situ from the videos were based on the in vivo morphology of the 30 specimens of *M. interjecta* collected from the ROV and submersible dives from 96 m to 278 m depth (Table 2). These collections allowed us to confidently identify the species from the video footage 392 times, from 83 to 280 m. This depth range is in accordance with previous records for this species (Fricke and Hottinger 1983; Scheer and Pillai 1983).

*M. interjecta* was recorded in 44% of transects (15 out of 34) in the Gulf of Aqaba, which was the highest proportion of observations for any region. Some records in the northern Red Sea and Gulf of Aqaba may be repeated, due to the overlap of sites between expeditions, for example, the bioherm in the northern Red Sea was observed on three transects. However, although *M. interjecta* was observed in a range of habitats (Figure 6), it was more frequently recorded on steep to vertical seafloor, which is common in the Gulf of Aqaba and northern Red Sea (Purkis et al. 2022). This habitat may have been inconsistently sampled in other regions of the basin, particularly in the central Red Sea, where the seafloor is not defined by steep slopes, and *M. interjecta* was only observed twice in 36 dives (Figure 3a). *M. interjecta* was identified in over a quarter of transects in the southern Red Sea (28%), and the high occurrence rate here may be due to the morphological diversity of Difaht Farasan (also known as the Farasan Bank; Rowlands et al. 2016).

*M. interjecta* bioherms were encountered at three sites throughout our study region (Figure 1a,b; Figure 3), extending the known occurrence of these features in the Red Sea beyond the Gulf of Aqaba (Fricke and Hottinger 1983). The largest of these bioherms was found in the northern Gulf of Aqaba, where the highest density of *M. interjecta* observations (119) was recorded. These bioherms provide substrate for a diverse range of benthic taxa, particularly in the lower mesophotic. Moreover, their three‐dimensional and often complex structure creates shelter for fish (Figure 1a,b). The bioherms studied here play a key ecosystem structuring role, comparable to that of many deep corals worldwide. Two of the most widely distributed deep constructional corals are *Desmophyllum pertusum* (Linnaeus, 1758) and *Madrepora oculata* Linnaeus, 1758, both of which are present from the Mediterranean (Chimienti et al. 2018; Fanelli et al. 2017; Matos et al. 2021) to the North Atlantic (Arnaud‐Haond et al. 2017) and Gulf of Mexico (Schroeder et al. 2005), and often seen co‐occurring (e.g., Arnaud‐Haond et al. 2017). Other species are also known to form deep coral bioherms, including *Solenosmilia variabilis* Duncan, 1873 in Brazil (Raddatz et al. 2019) and *Enallopsammia profunda* (Pourtalès, 1867) and *Oculina varicosa* Le Sueur, 1820 in Florida (Reed 2002; Reed et al. 2013). These deep species from outside the Red Sea are found in cooler temperatures but still support a high diversity of associated fauna, as seen here on *Madracis* bioherms (e.g., Ramos et al. 2017).

The areas identified as highly suitable for *M. interjecta* by the model covered a depth range of 273 m, slightly greater than that observed in this study (182 m) or reported previously (i.e., depth range of 230 m; Fricke and Hottinger 1983). While the lower depth limit of the models (295 m) agrees with observed values, the upper limit is much shallower at 22 m depth (Figure 6b). This is shallower than the depth range examined in the present study, so we cannot confirm the presence of the species at this depth. However, it is possible that the abiotic conditions are physiologically suitable for *Madracis interjecta* , but that biotic interactions, such as competition for space with zooxanthellate species (Kahng et al. 2010), would inhibit the distribution of the species into these shallower waters. Due to the relatively narrow bathymetric range, it is unsurprising to see depth among the strongest predictors. Furthermore, our finding is in agreement with several other published habitat suitability models that identified depth as the primary driver for the distribution of scleractinian habitat builders (e.g., Costa et al. 2015; Nolan et al. 2024; Tracey et al. 2011). Of the three environmental variables, dissolved oxygen concentration had the highest contribution to the model (6.8%; Table 1) and was the only environmental variable retained in the final model, perhaps due to the low, yet highly variable oxygen concentration in the northern Red Sea between 100 m and 400 m (Sofianos and Johns 2007). Dissolved oxygen concentration has been suggested to influence the formation of cold water coral mounds of Mauritania, albeit on different coral families (Caryophylliidae Dana, 1846 and Madreporidae Ehrenberg, 1834) (Wienberg et al. 2018). However, the locations of both *M. interjecta* colonies and bioherms had similar measurements for dissolved oxygen concentration in this study, suggesting it is unlikely to be a limiting factor to bioherm formation in the Red Sea.

Areas predicted to be highly suitable as *M. interjecta* habitat were seen throughout our study site, covering 65.84 km^2^. Notably, this is an area over ten times larger than the one estimated from similar models for Foraminiferal Algal Nodules (FANs), a mesophotic habitat in the same region of the Red Sea (Bracchi et al. 2023). The greater area that is suitable for *M. interjecta* when compared to FANs is facilitated by the wider, although overlapping, depth interval in which it was predicted to live (340 m for *M. interjecta* vs. 73 m for FANs). In contrast, the suitable area predicted here for *M. interjecta* was lower than the estimated area suitable for deep coral frameworks built by corals of the families Caryophylliidae (> 100 km^2^) or Dendrophylliidae (> 150 km^2^) across the same area (Nolan et al. 2024). The average depth distribution of these coral frameworks is deeper than for *M. interjecta* , although they again overlap. Competition for space decreases with depth (Kahng and Kelley 2007), presenting a potential explanation for the ability of these deeper frameworks to expand over larger areas than *Madracis* bioherms. Nevertheless, these comparisons highlight the potential of *M. interjecta* colonies and bioherms as an important component of the Red Sea carbonate factory and provide further evidence that this system is productive below the shallows of the Red Sea (Heiss 1995; Serrano et al. 2018).

The model performed well according to all metrics (AUC and TSS), providing us with a high degree of confidence in the above results. Despite this, there are a few limitations to consider in our study. We estimate the potential distribution based on abiotic variables that could be obtained during the expedition, yet the distribution of *M. interjecta* may in fact be driven by other variables. For example, the azooxanthellate coral *Dendrophyllia cornigera* (Lamarck, 1816) has been shown to capture more macrozooplankton under higher flow speeds (Gori et al. 2015), suggesting that other azooxanthellate corals, such as *M. interjecta* , may prefer to settle in areas with higher current velocities for increased food availability. Furthermore, currents have been linked to the presence and form of deep coral reefs (Sanna et al. 2023; van der Kaaden et al. 2023), so they may influence the location of *Madracis* bioherms. While we could not directly include current velocity here, we have included measures of seafloor complexity, which influence and can act as a proxy for current velocities (Purkis and Kohler 2008). Uncertainties are inherent in modelling but may be confounded by survey bias, where transects are not conducted following true randomisation, and a subset of the population in question is unintentionally excluded (Phillips et al. 2009; Vierod et al. 2014). This becomes a problem when the excluded data is correlated with the predictor variables. Although we attempted to reduce bias through widely dispersed survey transects (Figure 3), the exploratory nature of the expeditions and transects indicates that this small bias may remain and represents a limitation in our dataset. The uncertainty in our model is represented as the standard deviation between model repeats (Ibáñez et al. 2009; Figure 5). The highest values of uncertainty coincide with areas that are, on average, estimated to be highly suitable, suggesting that while the model repeats are generally in agreement on where the model is not suitable, there is more discrepancy in where it is highly suitable. However, these values remain low, with a maximum of 0.27 (Figure 5). Some evaluation metrics, such as TSS, require the selection of a threshold value in their calculation. While previous studies have chosen to report threshold‐independent validation metrics to avoid the introduction of potential errors through threshold selection (Vierod et al. 2014), we report both the AUC and TSS. As suggested by Liu et al. (2016), we used the maximised sum of sensitivity and specificity (MaxSSS), where sensitivity refers to the proportion of positives that are true positives (i.e., areas which are accurately predicted to contain presences) and specificity is the proportion of absences that are true absences (i.e., areas which are correctly predicted to be unsuitable). This method is preferred due to its objective selection, its applicability to both presence‐absence and presence‐only data, and its ability to discriminate between presences and absences (Liu et al. 2016).

The limits in a coral species' distribution are based on both biotic and abiotic factors (Eyal et al. 2016; Ziegler et al. 2014). Compared to the actual distribution of the foundation framework‐building species that form them, coral bioherms occur in a narrower range of environmental conditions for most environmental parameters both in shallow and deep water (e.g., Benzoni et al. 2003; Howell et al. 2011). This is likely due to limitations in the conditions under which bioherms can *de facto* form. Moreover, it is known that environmental conditions, such as light and nutrient levels, can change the growth form of scleractinian corals (Todd 2008), and *Madracis* colonies specifically (Bruno and Edmunds 1997; Filatov et al. 2010). Here, we observed *M. interjecta* bioherms occupying similar environmental niches to the individual species colonies, but with some notable differences (Figure 6). With a mean depth of 200 m, *M. interjecta* bioherms in the Red Sea occurred slightly deeper than individual colonies (mean = 160 m; Figure 6b). Additionally, all bioherms were observed at temperatures below 21.9°C, lower than the average temperature for colonies (Figure 6j), but comparable to the conditions of the *M. interjecta* bioherms reported by Fricke and Hottinger (1983) in the Gulf of Aqaba. For comparison, *M. myriaster* bioherms in Colombia develop at depths of 150–160 m (Alonso et al. 2021; Cedeño‐Posso et al. 2022) where nearby temperatures have been reported around 13°C–15°C (Woce Upper Ocean Thermal 2006). This record is both slightly shallower and at much lower temperatures than the colonies observed in the Red Sea, where the unique environmental conditions at depth have likely shaped the evolution and depth distribution of its marine biota and led to its high endemism rate (Dibattista et al. 2016; Türkay 1996). On the other hand, *Madracis* formations have been observed between 28 m and 44 m water depth in the north‐west Gulf of Mexico, in temperatures that vary annually between 18°C and 30°C (Schmahl et al. 2008). These two examples highlight the variability of tolerance exhibited within the *Madracis* genus. Additionally, the example from the Gulf of Mexico experiences a large thermal range between seasons (Schmahl et al. 2008). Our results suggest that there is, on average, no significant difference in the environmental conditions experienced by *M. interjecta* between seasons in the Red Sea (Figure 7b). However, to fully address the potential influence of seasonal variability on the distribution of *M. interjecta* , we would need to record environmental conditions at the same location in different seasons, in order to include the range, rather than the mean, as a predictor variable.

The PCA analysis (Figure 6a) grouped *M. interjecta* observations by region. Bioherm observations clustered together by site, but those in the northern Red Sea were separated from those in the Gulf of Aqaba, reflecting the differences in environmental conditions between these two regions (Yao et al. 2014). Unfortunately, not all variables were measured for the central Red Sea bioherm, and its observations could therefore not be included in this analysis. As this bioherm was observed much further south, the inclusion of such data may expand the range of conditions in which bioherms are known to form. Furthermore, additional variables may be required to understand these differences in full. For example, current is known to strongly influence the formation of coral mounds (van der Kaaden et al. 2021). While orientation variables (Northness and Eastness), included here, can act as a proxy for current velocity (Dolan et al. 2008), we did not have direct measurements of oceanographic currents to include in this study.

Here, we provide the first habitat suitability model for a mesophotic and deep‐water endemic coral in the Red Sea, indicating the extent of the potential distribution and highlighting its importance as a bioherm‐forming species. In light of ongoing coastal development around the Red Sea, and in particular the northern Red Sea and Gulf of Aqaba, there are several threats that may impact these species. Large‐scale coastal development impacts adjacent marine environments, for example through sedimentation (Maragos 1993; Stender et al. 2014), which may then be transported to the mesophotic or deep. Particularly the narrow shelf and steep slopes of the Gulf of Aqaba (Purkis et al. 2022; Weinstein et al. 2021) may result in greater or faster sedimentation at depth. Slow life histories make deep‐water corals particularly vulnerable to heavy sedimentation, which has been shown elsewhere to significantly impair survival (Brooke et al. 2009) or cause coral polyp mortality (Mobilia et al. 2023). As well as sediment, pollutants such as nitrogen may be discharged into the ocean during construction works, the effect of which remains unclear and variable on shallow reefs (Zhao et al. 2021), and unstudied on deep reefs. The limited understanding of the potential responses of deep Red Sea corals like *M. interjecta* highlights the need for a better ecological understanding, as well as baseline data on species abundance to directly inform marine conservation planning. The presence of *M. myriaster* as a unique primary habitat‐forming species prompted the formation of a protected area in Colombia (Alonso et al. 2021; Cedeño‐Posso et al. 2022), indicating their value globally. Finally, this work also provides a starting point for further studies into the mechanisms required for *M. interjecta* and other framework‐building species to form extensive bioherms.

## Author Contributions

**Megan K. B. Nolan:** conceptualization (equal), data curation (equal), formal analysis (lead), investigation (equal), methodology (lead), visualization (lead), writing – original draft (lead), writing – review and editing (equal). **Pauline Falkenberg:** data curation (equal), formal analysis (equal). **Fabio Marchese:** conceptualization (equal), data curation (equal), investigation (equal), supervision (equal), writing – review and editing (equal). **Marta A. Ezeta Watts:** data curation (equal), investigation (supporting), writing – review and editing (equal). **Natalie Dunn:** data curation (equal), investigation (supporting), writing – review and editing (equal). **Laura Macrina:** investigation (supporting), writing – review and editing (equal). **Viktor Nunes‐Peinemann:** investigation (supporting). **Giovanni Chimienti:** investigation (supporting), writing – review and editing (equal). **Silvia Vimercati:** investigation (supporting), writing – review and editing (equal). **Tullia I. Terraneo:** investigation (supporting). **Mohammed Qurban:** funding acquisition (equal), project administration (equal), resources (equal). **Vincent Pieribone:** investigation (supporting), project administration (equal), resources (equal). **Carlos M. Duarte:** funding acquisition (equal), investigation (supporting), project administration (equal), resources (equal), writing – review and editing (equal). **Francesca Benzoni:** conceptualization (equal), formal analysis (equal), funding acquisition (equal), investigation (supporting), supervision (equal), visualization (equal), writing – review and editing (equal).

## Conflicts of Interest

The authors declare no conflicts of interest.

## Data Availability

Data used in this study is available in the methods and Supporting Information of this paper. Additionally, environmental data layers used to generate the models are already published at https://doi.org/10.5281/zenodo.13935238.

## References

[ece371456-bib-0001] Allouche, O. , A. Tsoar , and R. Kadmon . 2006. “Assessing the Accuracy of Species Distribution Models: Prevalence, Kappa and the True Skill Statistic (TSS).” Journal of Applied Ecology 43, no. 6: 1223–1232. 10.1111/j.1365-2664.2006.01214.x.

[ece371456-bib-0002] Alonso, D. , M. Vides‐Casado , F. Arias‐Isaza , et al. 2021. “Behind the Scenes for the Designation of the Corales de Profundidad National Natural Park of Colombia.” Frontiers in Marine Science 8: 1–15. 10.3389/fmars.2021.567438.

[ece371456-bib-0003] Armstrong, R. A. , O. Pizarro , and C. Roman . 2019. Underwater Robotic Technology for Imaging Mesophotic Coral Ecosystems. Vol. 12, 973–988. Coral Reefs of the World. 10.1007/978-3-319-92735-0_51.

[ece371456-bib-0004] Arnaud‐Haond, S. , I. M. J. Van den Beld , R. Becheler , et al. 2017. “Two “Pillars” of Cold‐Water Coral Reefs Along Atlantic European Margins: Prevalent Association of *Madrepora oculata* With *Lophelia pertusa* , From Reef to Colony Scale.” Deep Sea Research Part II: Topical Studies in Oceanography 145: 110–119. 10.1016/j.dsr2.2015.07.013.

[ece371456-bib-0005] Asaad, I. , C. J. Lundquist , M. V. Erdmann , and M. J. Costello . 2018. “Delineating Priority Areas for Marine Biodiversity Conservation in the Coral Triangle.” Biological Conservation 222: 198–211. 10.1016/j.biocon.2018.03.037.

[ece371456-bib-0006] Augustin, N. , F. M. van der Zwan , C. W. Devey , and B. Brandsdóttir . 2021. “13 Million Years of Seafloor Spreading Throughout the Red Sea Basin.” Nature Communications 12, no. 1: 1–10. 10.1038/s41467-021-22586-2. PMC8065172 33893306

[ece371456-bib-0007] Bargain, A. , F. Marchese , A. Savini , M. Taviani , and M.‐C. Fabri . 2017. “Santa Maria di Leuca Province (Mediterranean Sea): Identification of Suitable Mounds for Cold‐Water Coral Settlement Using Geomorphometric Proxies and Maxent Methods.” Frontiers in Marine Science 4: 338. 10.3389/fmars.2017.00338.

[ece371456-bib-0008] Benzoni, F. , R. Arrigoni , M. L. Berumen , M. Taviani , P. Bongaerts , and P. R. Frade . 2018. “Morphological and Genetic Divergence Between Mediterranean and Caribbean Populations of *Madracis Pharensis* (Heller 1868) (Scleractinia, Pocilloporidae): Too Much for One Species?” Zootaxa 4471, no. 3: 473–492. 10.11646/zootaxa.4471.3.3. 30313392

[ece371456-bib-0009] Benzoni, F. , C. N. Bianchi , and C. Morri . 2003. “Coral Communities of the Northwestern Gulf of Aden (Yemen): Variation in Framework Building Related to Environmental Factors and Biotic Conditions.” Coral Reefs 22, no. 4: 475–484. 10.1007/s00338-003-0342-1.

[ece371456-bib-0010] Berumen, M. L. , M. B. Roberts , T. H. Sinclair‐Taylor , et al. 2019. “Fishes and Connectivity of Red Sea Coral Reefs.” In Coral Reefs of the Red Sea, 157–179. Springer. 10.1007/978-3-030-05802-9_8.

[ece371456-bib-0011] Berumen, M. L. , C. R. Voolstra , D. Daffonchio , et al. 2019. “The Red Sea: Environmental Gradients Shape a Natural Laboratory in a Nascent Ocean.” In Coral Reefs of the Red Sea, 1–10. Springer International Publishing.

[ece371456-bib-0012] Bongaerts, P. , T. Ridgway , E. M. Sampayo , and O. Hoegh‐Guldberg . 2010. “Assessing the ‘Deep Reef Refugia’ Hypothesis: Focus on Caribbean Reefs.” Coral Reefs 29, no. 2: 309–327. 10.1007/s00338-009-0581-x.

[ece371456-bib-0013] Bozinovic, F. , P. Calosi , and J. I. Spicer . 2011. “Physiological Correlates of Geographic Range in Animals.” Annual Review of Ecology, Evolution, and Systematics 42, no. 1: 155–179. 10.1146/annurev-ecolsys-102710-145055.

[ece371456-bib-0014] Bracchi, V. A. , S. J. Purkis , F. Marchese , et al. 2023. “Mesophotic Foraminiferal‐Algal Nodules Play a Role in the Red Sea Carbonate Budget.” Communications Earth & Environment 4, no. 1: 288. 10.1038/s43247-023-00944-w.

[ece371456-bib-0015] Brooke, S. D. , M. W. Holmes , and C. M. Young . 2009. “Sediment Tolerance of Two Different Morphotypes of the Deep‐Sea Coral *Lophelia pertusa* From the Gulf of Mexico.” Marine Ecology Progress Series 390: 137–144. 10.3354/meps08191.

[ece371456-bib-0016] Bruno, J. F. , and P. J. Edmunds . 1997. “Clonal Variation for Phenotypic Plasticity in the Coral *Madracis Mirabilis* .” Ecology 78, no. 7: 2177–2190. 10.1890/0012-9658.

[ece371456-bib-0017] Cairns, S. D. 2000. “A Revision of the Shallow‐Water Azooxanthellate Scleractinia of the Western Atlantic.” Studies on the Natural History of the Caribbean Region 75: 1–231.

[ece371456-bib-0018] Cedeño‐Posso, C. , M. Vides‐Casado , V. Rocha , G. H. Borrero‐Pérez , A. Polanco F , and D. Alonso . 2022. “Benthic Macrohabitat Classification and Madracis Spp. Coral Patch Distribution in a Deep‐Sea Marine Protected Area of Colombia.” Frontiers in Marine Science 9: 995419. 10.3389/fmars.2022.995419.

[ece371456-bib-0019] Chimienti, G. , M. Bo , and F. Mastrototaro . 2018. “Know the Distribution to Assess the Changes: Mediterranean Cold‐Water Coral Bioconstructions.” Rendiconti Lincei. Scienze Fisiche e Naturali 29, no. 3: 583–588. 10.1007/s12210-018-0718-3.

[ece371456-bib-0020] Core Development Team, R . 2020. A Language and Environment for Statistical Computing. R Foundation for Statistical Computing R Foundation for Statistical Computing. http://www.r‐project.org.

[ece371456-bib-0021] Costa, B. , M. S. Kendall , F. A. Parrish , et al. 2015. “Identifying Suitable Locations for Mesophotic Hard Corals Offshore of Maui, Hawai'i.” PLoS One 10, no. 7: e0130285. 10.1371/journal.pone.0130285. 26153883 PMC4495987

[ece371456-bib-0022] Crowder, L. , and E. Norse . 2008. “Essential Ecological Insights for Marine Ecosystem‐Based Management and Marine Spatial Planning.” Marine Policy 32, no. 5: 772–778. 10.1016/j.marpol.2008.03.012.

[ece371456-bib-0023] da Silveira, C. B. L. , G. M. R. Strenzel , M. Maida , A. L. B. Gaspar , and B. P. Ferreira . 2021. “Coral Reef Mapping With Remote Sensing and Machine Learning: A Nurture and Nature Analysis in Marine Protected Areas.” Remote Sensing 13, no. 15: 2907. 10.3390/rs13152907.

[ece371456-bib-0024] De Clippele, L. H. , L. Rovelli , B. Ramiro‐Sánchez , et al. 2020. “Mapping Cold‐Water Coral Biomass: An Approach to Derive Ecosystem Functions.” Coral Reefs 40, no. 1: 215–231. 10.1007/s00338-020-02030-5.

[ece371456-bib-0025] Dibattista, J. D. , J. Howard Choat , M. R. Gaither , et al. 2016. “On the Origin of Endemic Species in the Red Sea.” Journal of Biogeography 43, no. 1: 13–30. 10.1111/jbi.12631.

[ece371456-bib-0026] Diekmann, O. E. 2003. The Coral Genus Madracis. Speciation in Corals and Their Symbionts. Thesis, Universiteit van Amsterdam.

[ece371456-bib-0027] Dolan, M. F. J. , A. J. Grehan , J. C. Guinan , and C. Brown . 2008. “Modelling the Local Distribution of Cold‐Water Corals in Relation to Bathymetric Variables: Adding Spatial Context to Deep‐Sea Video Data.” Deep‐Sea Research Part I: Oceanographic Research Papers 55, no. 11: 1564–1579. 10.1016/J.DSR.2008.06.010.

[ece371456-bib-0028] Elith, J. , C. H. Graham , R. P. Anderson , et al. 2006. “Novel Methods Improve Prediction of Species' Distributions From Occurrence Data.” Ecography 29, no. 2: 129–151. 10.1111/J.2006.0906-7590.04596.X.

[ece371456-bib-0029] Elith, J. , and J. R. Leathwick . 2009. “Species Distribution Models: Ecological Explanation and Prediction Across Space and Time.” Annual Review of Ecology, Evolution, and Systematics 40, no. 1: 677–697. 10.1146/annurev.ecolsys.110308.120159.

[ece371456-bib-0030] Elith, J. , J. R. Leathwick , and T. Hastie . 2008. “A Working Guide to Boosted Regression Trees.” Journal of Animal Ecology 77, no. 4: 802–813. 10.1111/j.1365-2656.2008.01390.x. 18397250

[ece371456-bib-0031] Enrichetti, F. , M. Bo , C. Morri , et al. 2019. “Assessing the Environmental Status of Temperate Mesophotic Reefs: A New, Integrated Methodological Approach.” Ecological Indicators 102: 218–229. 10.1016/j.ecolind.2019.02.028.

[ece371456-bib-0032] Evans, T. G. , S. E. Diamond , and M. W. Kelly . 2015. “Mechanistic Species Distribution Modelling as a Link Between Physiology and Conservation.” Conservation Physiology 3, no. 1: cov056. 10.1093/conphys/cov056. 27293739 PMC4778482

[ece371456-bib-0033] Eyal, G. , L. Eyal‐Shaham , I. Cohen , et al. 2016. “ *Euphyllia Paradivisa*, a Successful Mesophotic Coral in the Northern Gulf of Eilat/Aqaba, Red Sea.” Coral Reefs 35, no. 1: 91–102. 10.1007/s00338-015-1372-1.

[ece371456-bib-0034] Fanelli, E. , I. Delbono , R. Ivaldi , M. Pratellesi , S. Cocito , and A. Peirano . 2017. “Cold‐Water Coral *Madrepora Oculata* in the Eastern Ligurian Sea (NW Mediterranean): Historical and Recent Findings.” Aquatic Conservation: Marine and Freshwater Ecosystems 27, no. 5: 965–975. 10.1002/aqc.2751.

[ece371456-bib-0035] Filatov, M. V. , J. A. Kaandorp , M. Postma , et al. 2010. “A Comparison Between Coral Colonies of the Genus Madracis and Simulated Forms.” Proceedings of the Biological Sciences 277, no. 1700: 3555–3561. 10.1098/rspb.2010.0957. 20573621 PMC2982250

[ece371456-bib-0036] Fricke, H. W. , and L. Hottinger . 1983. “Coral Bioherms Below the Euphotic Zone in the Red Sea.” Marine Ecology Progress Series 11: 113–117.

[ece371456-bib-0037] Fricke, H. W. , and B. Knauer . 1986. “Diversity and Spatial Pattern of Coral Communities in the Red Sea Upper Twilight Zone.” Oecologia 71, no. 1: 29–37. 10.1007/BF00377316. 28312079

[ece371456-bib-0038] Gori, A. , S. Reynaud , C. Orejas , and C. Ferrier‐Pagès . 2015. “The Influence of Flow Velocity and Temperature on Zooplankton Capture Rates by the Cold‐Water Coral *Dendrophyllia cornigera* .” Journal of Experimental Marine Biology and Ecology 466: 92–97. 10.1016/j.jembe.2015.02.004.

[ece371456-bib-0039] Greene, H. G. , M. M. Yoklavich , R. M. Starr , et al. 1999. “A Classification Scheme for Deep Seafloor Habitats.” Oceanologica Acta 22, no. 6: 663–678. 10.1016/S0399-1784(00)88957-4.

[ece371456-bib-0040] Harley, C. D. , A. Randall Hughes , K. M. Hultgren , et al. 2006. “The Impacts of Climate Change in Coastal Marine Systems.” Ecology Letters 9, no. 2: 228–241. 10.1111/j.1461-0248.2005.00871.x. 16958887

[ece371456-bib-0041] Hastings, R. A. , L. A. Rutterford , J. J. Freer , R. A. Collins , S. D. Simpson , and M. J. Genner . 2020. “Climate Change Drives Poleward Increases and Equatorward Declines in Marine Species.” Current Biology 30, no. 8: 1572–1577. 10.1016/j.cub.2020.02.043. 32220327

[ece371456-bib-0042] Heiss, G. A. 1995. “Carbonate Production by Scleractinian Corals at Aqaba, Gulf of Aqaba, Red Sea.” Facies 33: 19–34.

[ece371456-bib-0043] Horn, B. K. P. 1981. “Hill Shading and the Reflectance Map.” Proceedings of the IEEE 69, no. 1: 14–47.

[ece371456-bib-0044] Howell, K. L. , R. Holt , I. P. Endrino , and H. Stewart . 2011. “When the Species Is Also a Habitat: Comparing the Predictively Modelled Distributions of Lophelia Pertusa and the Reef Habitat It Forms.” Biological Conservation 144, no. 11: 2656–2665. 10.1016/J.BIOCON.2011.07.025.

[ece371456-bib-0045] Ibáñez, I. , J. A. Silander Jr. , A. M. Wilson , N. LaFleur , N. Tanaka , and I. Tsuyama . 2009. “Multivariate Forecasts of Potential Distributions of Invasive Plant Species.” Ecological Applications 19, no. 2: 359–375. 10.1890/07-2095.1. 19323195

[ece371456-bib-0046] Ingrosso, G. , M. Abbiati , F. Badalamenti , et al. 2018. “Mediterranean Bioconstructions Along the Italian Coast.” Advances in Marine Biology 79: 61–136. 10.1016/bs.amb.2018.05.001. 30012277

[ece371456-bib-0047] Iwahashi, J. , and R. J. Pike . 2007. “Automated Classifications of Topography From DEMs by an Unsupervised Nested‐Means Algorithm and a Three‐Part Geometric Signature.” Geomorphology 86, no. 3–4: 409–440. 10.1016/J.GEOMORPH.2006.09.012.

[ece371456-bib-0048] Iwatsuki, Y. , T. Matsuda , W. C. Starnes , T. Nakabo , and T. Yoshino . 2012. “A Valid Priacanthid Species, Pristigenys Refulgens (Valenciennes 1862), and a Redescription of *P. Niphonia* (Cuvier in Cuvier & Valenciennes 1829) in the Indo‐West Pacific (Perciformes: Priacanthidae).” Zootaxa 3206, no. 1: 41–57. 10.11646/zootaxa.3206.1.2.

[ece371456-bib-0049] Jamieson, A. J. , G. Singleman , T. D. Linley , and S. Casey . 2020. “Fear and Loathing of the Deep Ocean: Why Don't People Care About the Deep Sea?” ICES Journal of Marine Science 78, no. 3: 797–809. 10.1093/icesjms/fsaa234.

[ece371456-bib-0050] Jenness, J. S. 2004. “Calculating Landscape Surface Area From Digital Elevation Models.” Wildlife Society Bulletin 32, no. 3: 829–839. 10.2193/0091-7648(2004)032[0829:Clsafd]2.0.Co;2.

[ece371456-bib-0051] Kahng, S. E. , J. M. Copus , and D. Wagner . 2014. “Recent Advances in the Ecology of Mesophotic Coral Ecosystems (MCEs).” Current Opinion in Environmental Sustainability 7: 72–81. 10.1016/J.COSUST.2013.11.019.

[ece371456-bib-0052] Kahng, S. E. , J. R. Garcia‐Sais , H. L. Spalding , et al. 2010. “Community Ecology of Mesophotic Coral Reef Ecosystems.” Coral Reefs 29, no. 2: 255–275. 10.1007/s00338-010-0593-6.

[ece371456-bib-0053] Kahng, S. E. , and C. D. Kelley . 2007. “Vertical Zonation of Megabenthic Taxa on a Deep Photosynthetic Reef (50–140 m) in the Au'au Channel, Hawaii.” Coral Reefs 26, no. 3: 679–687. 10.1007/s00338-007-0253-7.

[ece371456-bib-0054] Kassambara, A. , and F. Mundt . 2020. “Factoextra: Extract and Visualize the Results of Multivariate Data Analyses.” https://cran.r‐project.org/web/packages/factoextra/readme/README.html.

[ece371456-bib-0055] Koethe, R. , and F. Lehmeier . 1996. “SARA—System Zur Automatischen Relief‐Analyse [User Manual].”

[ece371456-bib-0056] Kramer, N. , G. Eyal , R. Tamir , and Y. Loya . 2019. “Upper Mesophotic Depths in the Coral Reefs of Eilat, Red Sea, Offer Suitable Refuge Grounds for Coral Settlement.” Scientific Reports 9, no. 1: 1–12. 10.1038/s41598-019-38795-1. 30783139 PMC6381148

[ece371456-bib-0057] La Bianca, G. , S. Rees , M. J. Attrill , et al. 2023. “A Standardised Ecosystem Services Framework for the Deep Sea.” Frontiers in Marine Science 10: 1176230. 10.3389/fmars.2023.1176230.

[ece371456-bib-0058] Lê, S. , J. Josse , and F. Husson . 2008. “FactoMineR: A Package for Multivariate Analysis.” Journal of Statistical Software 25, no. 1: 1–18. 10.18637/jss.v025.i01.

[ece371456-bib-0059] Liu, C. , G. Newell , and M. White . 2016. “On the Selection of Thresholds for Predicting Species Occurrence With Presence‐Only Data.” Ecology and Evolution 6, no. 1: 337–348. 10.1002/ece3.1878. 26811797 PMC4716501

[ece371456-bib-0060] Loya, Y. , G. Eyal , T. Treibitz , M. P. Lesser , and R. Appeldoorn . 2016. “Theme Section on Mesophotic Coral Ecosystems: Advances in Knowledge and Future Perspectives.” Coral Reefs 35, no. 1: 1–9. 10.1007/S00338-016-1410-7.

[ece371456-bib-0061] Maragos, J. E. 1993. “Impact of Coastal Construction on Coral Reefs in the U.S.‐Affiliated Pacific Islands.” Coastal Management 21, no. 4: 235–269. 10.1080/08920759309362207.

[ece371456-bib-0062] Marenzeller, E. V. 1907. “Expedition SM Schiff ‘Pola’ in Das Rote Meer, Nördliche und südliche Hälfte 1895.”

[ece371456-bib-0063] Martello, M. F. , J. Bleuel , M. G. Pennino , and G. O. Longo . 2024. “Projected Climate‐Driven Shifts in Coral Distribution Indicate Tropicalisation of Southwestern Atlantic Reefs.” Diversity and Distributions 30: e13851. 10.1111/ddi.13851.

[ece371456-bib-0064] Matos, F. L. , J. B. Company , and M. R. Cunha . 2021. “Mediterranean Seascape Suitability for *Lophelia pertusa* : Living on the Edge.” Deep Sea Research Part I: Oceanographic Research Papers 170: 103496. 10.1016/j.dsr.2021.103496.

[ece371456-bib-0065] McMannus, J. W. 2001. “Coral Reefs.” In Encyclopedia of Ocean Sciences, vol. 1, 524–534. Academic Press.

[ece371456-bib-0066] Merow, C. , M. J. Smith , and J. A. Silander . 2013. “A Practical Guide to MaxEnt for Modeling Species' Distributions: What It Does, and Why Inputs and Settings Matter.” Ecography 36, no. 10: 1058–1069. 10.1111/j.1600-0587.2013.07872.x.

[ece371456-bib-0067] Mobilia, V. , D. M. Tracey , V. Cummings , M. R. Clark , L. Woods , and J. Bell . 2023. “Effects of Sediment Pulses on the Deep‐Sea Coral *Goniocorella Dumosa* .” New Zealand Journal of Marine and Freshwater Research no. 1: 1–17. 10.1080/00288330.2023.2230154.

[ece371456-bib-0068] Morri, C. , D. Vafidis , A. Peirano , C. C. Chintiroglou , and C. N. Bianchi . 2000. “Anthozoa From a Subtidal Hydrothermal Area of Milos Island (Aegean Sea), With Notes on the Construction Potential of the Scleractinian Coral *Madracis Pharensis* .” Italian Journal of Zoology 67, no. 3: 319–325. 10.1080/11250000009356331.

[ece371456-bib-0069] Nolan, M. K. B. , C. J. S. Kim , O. Hoegh‐Guldberg , and M. Beger . 2021. “The Benefits of Heterogeneity in Spatial Prioritisation Within Coral Reef Environments.” Biological Conservation 258: 109155. 10.1016/j.biocon.2021.109155.

[ece371456-bib-0070] Nolan, M. K. B. , F. Marchese , S. J. Purkis , et al. 2024. “Habitat Suitability Models Reveal Extensive Distribution of Deep Warm‐Water Coral Frameworks in the Red Sea.” Communications Earth & Environment 5, no. 1: 709. 10.1038/s43247-024-01830-9.

[ece371456-bib-0071] Oksanen, J. , G. L. Simpson , F. G. Blanchet , et al. 2024. “Vegan: Community Ecology Package. In R Package Version 2.3‐5.”

[ece371456-bib-0072] Phillips, S. J. , M. Dudik , J. Elith , et al. 2009. “Sample Selection Bias and Presence‐Only Distribution Models: Implications for Background and Pseudo‐Absence Data.” Ecological Applications 19, no. 1: 181–197. 10.1890/07-2153.1. 19323182

[ece371456-bib-0073] Pinsky, M. L. , B. Worm , M. J. Fogarty , J. L. Sarmiento , and S. A. Levin . 2013. “Marine Taxa Track Local Climate Velocities.” Science 341, no. 6151: 1239–1242. 10.1126/science.1239352. 24031017

[ece371456-bib-0074] Purkis, S. J. , and K. E. Kohler . 2008. “The Role of Topography in Promoting Fractal Patchiness in a Carbonate Shelf Landscape.” Coral Reefs 27, no. 4: 977–989. 10.1007/s00338-008-0404-5.

[ece371456-bib-0075] Purkis, S. J. , H. Shernisky , P. K. Swart , et al. 2022. “Discovery of the Deep‐Sea NEOM Brine Pools in the Gulf of Aqaba, Red Sea.” Communications Earth & Environment 3, no. 1: 1–13. 10.1038/s43247-022-00482-x.

[ece371456-bib-0076] Pyle, R. L. 1996. “Exploring Deep Coral Reefs: How Much Biodiversity Are We Missing?” Global Biodiversity 6, no. 1: 3–7.

[ece371456-bib-0077] Raddatz, J. , J. Titschack , N. Frank , et al. 2019. “ *Solenosmilia Variabilis*‐Bearing Cold‐Water Coral Mounds Off Brazil.” Coral Reefs 39, no. 1: 69–83. 10.1007/s00338-019-01882-w.

[ece371456-bib-0078] Ramos, A. , J. L. Sanz , F. Ramil , L. M. Agudo , and C. Presas‐Navarro . 2017. “The Giant Cold‐Water Coral Mounds Barrier Off Mauritania.” In Deep‐Sea Ecosystems Off Mauritania: Research of Marine Biodiversity and Habitats in the Northwest African Margin, 481–525. Springer Netherlands. 10.1007/978-94-024-1023-5_13.

[ece371456-bib-0079] Ramos, E. , J. R. Díaz de Terán , A. Puente , and J. A. Juanes . 2016. “The Role of Geomorphology in the Distribution of Intertidal Rocky Macroalgae in the NE Atlantic Region.” Estuarine, Coastal and Shelf Science 179: 90–98. 10.1016/j.ecss.2015.10.007.

[ece371456-bib-0080] Reed, J. K. 2002. “Deep‐Water Oculina Coral Reefs of Florida: Biology, Impacts, and Management.” Hydrobiologia 471: 43–55.

[ece371456-bib-0081] Reed, J. K. , C. Messing , B. K. Walker , et al. 2013. “Habitat Characterization, Distribution, and Areal Extent of Deep‐Sea Coral Ecosystems Off Florida, Southeastern U.S.A.” Caribbean Journal of Science 47, no. 1: 13–30. 10.18475/cjos.v47i1.a3.

[ece371456-bib-0082] Reyes, J. , N. Santodomingo , and P. Florez . 2010. Corales Escleractinios de Colombia. Invemar Serie de Publicaciones Especiales.

[ece371456-bib-0083] Roberts, J. M. 2009. Cold‐Water Corals: The Biology and Geology of Deep‐Sea Coral Habitats. Cambridge University Press.

[ece371456-bib-0084] Roberts, J. M. , L. A. Henry , D. Long , and J. P. Hartley . 2008. “Cold‐Water Coral Reef Frameworks, Megafaunal Communities and Evidence for Coral Carbonate Mounds on the Hatton Bank, North East Atlantic.” 54, no. 3: 297–316. 10.1007/s10347-008-0140-x.

[ece371456-bib-0085] Roder, C. , M. L. Berumen , J. Bouwmeester , E. Papathanassiou , A. Al‐Suwailem , and C. R. Voolstra . 2013. “First Biological Measurements of Deep‐Sea Corals From the Red Sea.” Scientific Reports 3: 2802. 10.1038/srep02802. 24091830 PMC3789407

[ece371456-bib-0086] Roncolato, F. , T. E. Fellowes , S. Duce , et al. 2024. “Ecomorphodynamics of Oyster Reefs and Their Influence on Oyster Reef Morphology.” Geomorphology 456: 109213. 10.1016/j.geomorph.2024.109213.

[ece371456-bib-0087] Rowlands, G. , S. Purkis , and A. Bruckner . 2016. “Tight Coupling Between Coral Reef Morphology and Mapped Resilience in the Red Sea.” Marine Pollution Bulletin 105, no. 2: 575–585. 10.1016/j.marpolbul.2015.11.027. 26621578

[ece371456-bib-0088] Sanna, G. , J. V. Büscher , and A. Freiwald . 2023. “Cold‐Water Coral Framework Architecture Is Selectively Shaped by Bottom Current Flow.” Coral Reefs 42, no. 2: 483–495. 10.1007/s00338-023-02361-z.

[ece371456-bib-0089] Santodomingo, N. , J. Reyes , A. Gracia , A. Martínez , G. Ojeda , and C. García . 2007. “Azooxanthellate *Madracis* Coral Communities Off San Bernardo and Rosario Islands (Colombian Caribbean).” Bulletin of Marine Science 81, no. 3: 273–287.

[ece371456-bib-0090] Sappington, J. M. , K. M. Longshore , and D. B. Thompson . 2007. “Quantifying Landscape Ruggedness for Animal Habitat Analysis: A Case Study Using Bighorn Sheep in the Mojave Desert.” Journal of Wildlife Management 71, no. 5: 1419–1426. 10.2193/2005-723.

[ece371456-bib-0091] Scheer, G. , and C. S. Pillai . 1983. “Report on the Stony Corals From the Red Sea.” Zoologica 45: 1–98.

[ece371456-bib-0092] Schlitzer, R. 2021. “odv.awi.de.” https://odv.awi.de/.

[ece371456-bib-0093] Schmahl, G. P. , E. L. Hickerson , and W. F. Precht . 2008. “Biology and Ecology of Coral Reefs and Coral Communities in the Flower Garden Banks Region, Northwestern Gulf of Mexico.” In Coral Reefs of the USA, edited by B. M. Riegl and R. E. Dodge , 221–261. Springer Netherlands.

[ece371456-bib-0094] Schroeder, W. W. , S. D. Brooke , J. B. Olson , B. Phaneuf , J. J. McDonough III , and P. Etnoyer . 2005. “Occurrence of Deep‐Water Lophelia Pertusa and *Madrepora oculata* in the Gulf of Mexico.” In Cold‐Water Corals and Ecosystems, edited by A. Freiwald and J. M. Roberts . Springer. 10.1007/3-540-27673-4_14.

[ece371456-bib-0095] Schuhmacher, H. , and H. Zibrowius . 1985. “What Is Hermatypic? A Redefinition of Ecological Groups in Corals and Other Organisms.” Coral Reefs 4: 1–9.

[ece371456-bib-0096] Serrano, O. , H. Almahasheer , C. M. Duarte , and X. Irigoien . 2018. “Carbon Stocks and Accumulation Rates in Red Sea Seagrass Meadows.” Scientific Reports 8, no. 1: 15037. 10.1038/s41598-018-33182-8. 30302026 PMC6177483

[ece371456-bib-0097] Soares, M. d. O. , J. T. d. Araújo , S. M. C. Ferreira , et al. 2020. “Why Do Mesophotic Coral Ecosystems Have to Be Protected?” Science of the Total Environment 726: 138456. 10.1016/j.scitotenv.2020.138456. 32481209

[ece371456-bib-0098] Sofianos, S. S. , and W. E. Johns . 2007. “Observations of the Summer Red Sea Circulation.” Journal of Geophysical Research: Oceans 112, no. 6: 1–20. 10.1029/2006JC003886.

[ece371456-bib-0099] Stender, Y. , P. L. Jokiel , and K. S. Rodgers . 2014. “Thirty Years of Coral Reef Change in Relation to Coastal Construction and Increased Sedimentation at Pelekane Bay, Hawai'i.” PeerJ 2: e300. 10.7717/peerj.300. 24688875 PMC3961130

[ece371456-bib-0100] Su, Y. O. , H. C. Lin , and H. C. Ho . 2022. “A New Cryptic Species of the Pineapple Fish Genus Monocentris (Family Monocentridae) From the Western Pacific Ocean, With Redescription of *M. Japonica* (Houttuyn, 1782).” Zootaxa 5189, no. 1: 180–203. 10.11646/zootaxa.5189.1.18. 37045192

[ece371456-bib-0101] Suggett, D. J. , and M. J. H. van Oppen . 2022. “Horizon Scan of Rapidly Advancing Coral Restoration Approaches for 21st Century Reef Management.” Emerging Topics in Life Sciences 6, no. 1: 125–136. 10.1042/ETLS20210240. 35119476 PMC9023016

[ece371456-bib-0102] Terraneo, T. I. , F. Benzoni , A. H. Baird , R. Arrigoni , and M. L. Berumen . 2019. “Morphology and Molecules Reveal Two New Species of Porites (Scleractinia, Poritidae) From the Red Sea and the Gulf of Aden.” Systematics and Biodiversity 17, no. 5: 491–508. 10.1080/14772000.2019.1643806.

[ece371456-bib-0103] Terraneo, T. I. , M. Ouhssain , C. B. Castano , et al. 2023. “From the Shallow to the Mesophotic: A Characterization of Symbiodiniaceae Diversity in the Red Sea NEOM Region.” Frontiers in Marine Science 10: 1077805. 10.3389/fmars.2023.1077805.

[ece371456-bib-0104] Thurber, A. R. , A. K. Sweetman , B. E. Narayanaswamy , D. O. B. Jones , J. Ingels , and R. L. Hansman . 2014. “Ecosystem Function and Services Provided by the Deep Sea.” Biogeosciences 11, no. 14: 3941–3963. 10.5194/bg-11-3941-2014.

[ece371456-bib-0105] Tittensor, D. P. , A. R. Baco , P. E. Brewin , et al. 2009. “Predicting Global Habitat Suitability for Stony Corals on Seamounts.” Journal of Biogeography 36, no. 6: 1111–1128. 10.1111/j.1365-2699.2008.02062.x.

[ece371456-bib-0106] Todd, P. A. 2008. “Morphological Plasticity in Scleractinian Corals.” Biological Reviews of the Cambridge Philosophical Society 83, no. 3: 315–337. 10.1111/j.1469-185x.2008.00045.x. 18979594

[ece371456-bib-0107] Tracey, D. M. , A. A. Rowden , K. A. Mackay , and T. Compton . 2011. “Habitat‐Forming Cold‐Water Corals Show Affinity for Seamounts in the New Zealand Region.” In Marine Ecology Progress Series, vol. 430, 1–22. National Institute of Water and Atmospheric Research. 10.3354/meps09164.

[ece371456-bib-0108] Türkay, M. 1996. “Composition of the Deep Red Sea Macro‐And Megabenthic Invertebrate Fauna.” In Deep‐Sea and Extreme Shallow Water Habitats: Affinities and Adaptations. Biosystematics and Ecology Series, edited by F. Uiblein , J. Ott , and M. Stachowitsch , vol. 11, 43–59. Österreichische Akademie der Wissenschaften.

[ece371456-bib-0109] van der Kaaden, A.‐S. , S. R. Maier , S. Chen , et al. 2024. “Building Your Own Mountain: The Effects, Limits, and Drawbacks of Cold‐Water Coral Ecosystem Engineering.” Biogeosciences 21, no. 4: 973–992. 10.5194/bg-21-973-2024.

[ece371456-bib-0110] van der Kaaden, A. S. , S. R. Maier , K. Siteur , et al. 2023. “Tiger Reefs: Self‐Organized Regular Patterns in Deep‐Sea Cold‐Water Coral Reefs.” Ecosphere 14, no. 10: e4654. 10.1002/ecs2.4654.

[ece371456-bib-0111] van der Kaaden, A. S. , C. Mohn , T. Gerkema , et al. 2021. “Feedbacks Between Hydrodynamics and Cold‐Water Coral Mound Development.” Deep Sea Research Part I: Oceanographic Research Papers 178: 103641. 10.1016/J.DSR.2021.103641.

[ece371456-bib-0112] Vaughan, D. , and T. Agardy . 2019. “Marine Protected Areas and Marine Spatial Planning—Allocation of Resource Use and Environmental Protection.” In Marine Protected Areas: Science, Policy and Management, 13–35. Elsevier. 10.1016/B978-0-08-102698-4.00002-2.

[ece371456-bib-0113] Vierod, A. D. T. , J. M. Guinotte , and A. J. Davies . 2014. “Predicting the Distribution of Vulnerable Marine Ecosystems in the Deep Sea Using Presence‐Background Models.” Deep Sea Research Part II: Topical Studies in Oceanography 99: 6–18. 10.1016/j.dsr2.2013.06.010.

[ece371456-bib-0114] Vimercati, S. , T. I. Terraneo , C. B. Castano , et al. 2024. “Consistent Symbiodiniaceae Community Assemblage in a Mesophotic‐Specialist Coral Along the Saudi Arabian Red Sea.” Frontiers in Marine Science 11: 1264175. 10.3389/fmars.2024.1264175.

[ece371456-bib-0115] Waller, R. G. , S. Goode , D. Tracey , J. Johnstone , and A. Mercier . 2023. “A Review of Current Knowledge on Reproductive and Larval Processes of Deep‐Sea Corals.” Marine Biology 170, no. 5: 58. 10.1007/s00227-023-04182-8.

[ece371456-bib-0116] Wang, Y. , D. E. Raitsos , G. Krokos , J. A. Gittings , P. Zhan , and I. Hoteit . 2019. “Physical Connectivity Simulations Reveal Dynamic Linkages Between Coral Reefs in the Southern Red Sea and the Indian Ocean.” Scientific Reports 9, no. 1: 16598. 10.1038/s41598-019-53126-0. 31719628 PMC6851178

[ece371456-bib-0117] Weinstein, D. K. , R. Tamir , N. Kramer , et al. 2021. “Mesophotic Reef Geomorphology Categorization, Habitat Identification, and Relationships With Surface Cover and Terrace Formation in the Gulf of Aqaba.” Geomorphology 379: 107548. 10.1016/j.geomorph.2020.107548.

[ece371456-bib-0118] Weiss, A. 2001. Topographic Position and Landforms Analysis. Poster Presentation. ESRI User Conference, San Diego, CA.

[ece371456-bib-0119] Wernberg, T. , M. S. Thomsen , J. K. Baum , et al. 2024. “Impacts of Climate Change on Marine Foundation Species.” Annual Review of Marine Science 16: 247–282. 10.1146/annurev-marine-042023-093037. 37683273

[ece371456-bib-0120] Wienberg, C. , J. Titschack , N. Frank , et al. 2020. “Deglacial Upslope Shift of NE Atlantic Intermediate Waters Controlled Slope Erosion and Cold‐Water Coral Mound Formation (Porcupine Seabight, Irish Margin).” Quaternary Science Reviews 237: 106310. 10.1016/j.quascirev.2020.106310.

[ece371456-bib-0121] Wienberg, C. , J. Titschack , A. Freiwald , et al. 2018. “The Giant Mauritanian Cold‐Water Coral Mound Province: Oxygen Control on Coral Mound Formation.” Quaternary Science Reviews 185: 135–152. 10.1016/j.quascirev.2018.02.012.

[ece371456-bib-0122] Wisz, M. S. , J. Pottier , W. D. Kissling , et al. 2013. “The Role of Biotic Interactions in Shaping Distributions and Realised Assemblages of Species: Implications for Species Distribution Modelling.” Biological Reviews of the Cambridge Philosophical Society 88, no. 1: 15–30. 10.1111/j.1469-185X.2012.00235.x. 22686347 PMC3561684

[ece371456-bib-0123] Woce Upper Ocean Thermal, U. O. T . 2006. Water Temperature XBT Profiles From Cruise NBCQ91 (SCQC). National Oceanographic Data Center, Silver Spring, PANGAEA. 10.1594/PANGAEA.370967.

[ece371456-bib-0124] Yao, F. , I. Hoteit , L. J. Pratt , et al. 2014. “Seasonal Overturning Circulation in the Red Sea: 1. Model Validation and Summer Circulation.” Journal of Geophysical Research: Oceans 119, no. 4: 2238–2262. 10.1002/2013JC009004.

[ece371456-bib-0125] Zevenbergen, L. W. , and C. R. Thorne . 2006. “Quantitative Analysis of Land Surface Topography.” Earth Surface Processes and Landforms 12, no. 1: 47–56. 10.1002/esp.3290120107.

[ece371456-bib-0126] Zhao, H. , M. Yuan , M. Strokal , et al. 2021. “Impacts of Nitrogen Pollution on Corals in the Context of Global Climate Change and Potential Strategies to Conserve Coral Reefs.” Science of the Total Environment 774: 145017. 10.1016/j.scitotenv.2021.145017.

[ece371456-bib-0127] Ziegler, M. , C. M. Roder , C. Büchel , and C. R. Voolstra . 2014. “Limits to Physiological Plasticity of the Coral *Pocillopora verrucosa* From the Central Red Sea.” Coral Reefs 33, no. 4: 1115–1129. 10.1007/s00338-014-1192-8.

